# Effects of oral activated charcoal on hyperphosphatemia and vascular calcification in Chinese patients with stage 3–4 chronic kidney disease

**DOI:** 10.1007/s40620-018-00571-1

**Published:** 2018-12-26

**Authors:** Ying Gao, Guiyun Wang, Yang Li, Chenxiao Lv, Zunsong Wang

**Affiliations:** 1 Weifang Medical University, Weifang, People’s Republic of China; 2 Department of Nephrology, Qianfoshan Hospital, Shandong University, No. 16766 Jingshi Road, Jinan, 250014 Shandong People’s Republic of China; 3 Department of Nephrology, Chengxin Hospital of Yuncheng, Heze, 274700 Shandong People’s Republic of China

**Keywords:** Chronic kidney disease (CKD), Hyperphosphatemia, Activated charcoal, Vascular calcification

## Abstract

**Background:**

The relationship between oral activated charcoal (OAC) and hyperphosphatemia and vascular calcification is not completely clear. We observed and recorded the effects of OAC on hyperphosphatemia and vascular calcification in stage 3–4 chronic kidney disease (CKD).

**Methods:**

In a randomized controlled study, we included 97 patients with stage 3–4 CKD. In the first phase of the experiment, the patients were randomly divided into the OAC group and placebo group. The endpoint of this phase was the development of hyperphosphatemia. The patients with hyperphosphatemia were selected into the second phase of the study. These patients underwent coronary artery multidetector computed tomography (MDCT) and were randomly divided into three groups: the OAC group, the calcium carbonate (CC) group and the lanthanum carbonate (LC) group.

**Results:**

The first and second phases of the experiment were followed for 12 months. In the first phase of the experiment, there was a statistically significant difference in the proportion of patients with hyperphosphatemia between the OAC and placebo groups (28.57% vs. 79.17%, X^2^ = 24.958, P = 0.000). In the second phase, the differences in coronary calcification score (CACS) between the OAC group, the CC group and the LC group were statistically significant (525.5 ± 104.2 vs 688.1 ± 183.7 vs 431.4 ± 122.5, P < 0.01).

**Conclusion:**

Oral activated charcoal effectively delays the onset of hyperphosphatemia in patients with chronic kidney disease. OAC appears to delay the development of vascular calcifications in stage 3–4 CKD patients.

**Electronic supplementary material:**

The online version of this article (10.1007/s40620-018-00571-1) contains supplementary material, which is available to authorized users.

## Introduction

In recent years, chronic kidney disease (CKD) has become a worldwide public health issue [1]. The main factors affecting the prognosis of patients with chronic kidney disease are its complications, including cardiovascular and cerebrovascular diseases, malnutrition, inflammation, atherosclerosis syndrome and anemia [2]. Therefore, the prevention and treatment of complications of CKD are important to improve its prognosis. Cardiovascular disease (CVD) is the most common complication of CKD and is the most common cause of death in patients with CKD. Vascular calcification (VC) is the main cause of high CVD mortality in CKD patients [3]. Activated charcoal is widely used as an adsorbent in many fields, but there is little research regarding the preventive effect of medical charcoal on hyperphosphatemia and vascular calcification in stage 3–4 CKD patients with normal serum calcium and phosphorus levels. Our experiment focused on these aspects of CKD patients. This study was divided into two phases. In the first phase, the patients were randomly divided into the OAC group and placebo group. The endpoint of this phase was the development of hyperphosphatemia. Patients with hyperphosphatemia were selected to enter the second phase of the study, during which we studied the impact of three different phosphate binder strategies on coronary calcification.

## Methods

### Subjects

All patients were recruited from inpatient and outpatient departments at Qianfoshan Hospital, Shandong Province, China from January 2013 to December 2016. Inclusion criteria were as follows: 18 years of age or older; clinical diagnosis (history, serum creatinine, renal ultrasound, laboratory tests, etc.) of stage 3–4 CKD (creatinine clearance 15–60 ml/min, calculated by the CKD-EPI formula); serum calcium, phosphorus, and PTH levels within the normal range (serum calcium: 8.4–9.6 mg/dl, serum phosphorus: 2.7–4.6 mg/dl, serum PTH: 15.0–65.0 pg/ml); good general condition; good understanding of disease status and patient’s own health condition; and reasonable ability to communicate.

The exclusion criteria were as follows: (1) serious infection, fever, cough and expectoration, sore throat, abdominal pain, diarrhea, skin soft tissue infection such as carbuncle, furuncle, and complete blood counts showing leukocyte count beyond the normal range (10 × 10^9^/L); (2) serious cardiovascular diseases, including chronic cardiac failure level 3 or above, as well as various arrhythmias; (3) severe anemia: hemoglobin lower than 60 g/L; hypoalbuminemia: albumin less than 30 g/L; and (4) tumor: pathological evidence of tumor, or clinical manifestations and tumor markers suggesting the possibility of tumor.

**Fig. 1:**
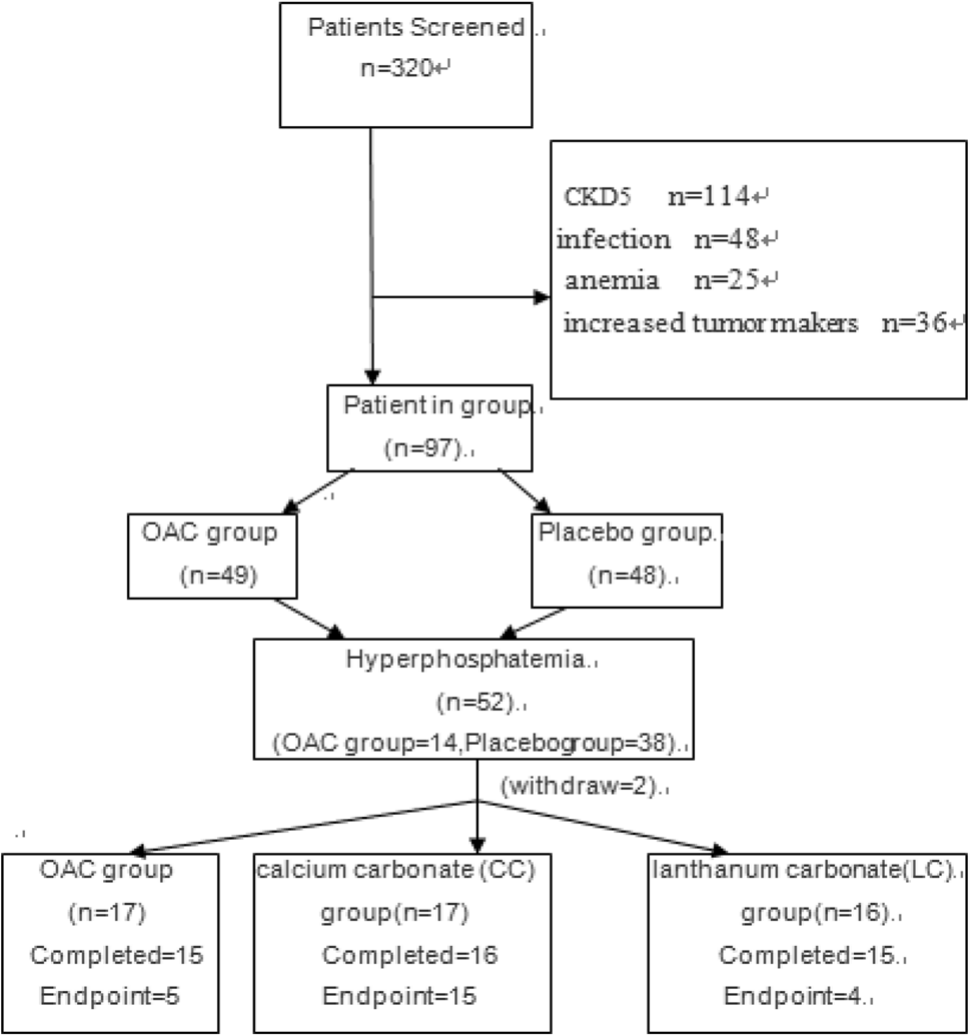
Patient flow chart

According to the above exclusion and inclusion criteria, 320 patients were screened. Some of the selected patients did not enter the trial eventually, and the reason is shown in Fig. 1. According to this test and the related formulas, we calculated that the sample size in the first stage is at least 52, and the sample size in the second stage is at least 45. Therefore, 97 patients were enrolled in the first stage of the study. Out of the 97 patients, 52 developed hyperphosphatemia by 12 months and 2 were lost to follow up. Therefore, 50 patients were enrolled into phase 2 of the study.

Written informed consent was obtained from all patients, and the study protocol was approved by the Human Research Ethics Committee of Qianfoshan Hospital (IRB approval protocol number 2012S025). The clinical trial registration number for this trial is ChiCTR1800018963.

### Experimental methods

The experimental period included two phases. During the first phase, all subjects were randomly divided into the OAC group or the placebo group. The OAC group was given oral activated charcoal (Changtian pharmaceuticals company, Hebei, China), 0.6–1.2 g each time, three times a day, taken with food. The placebo group was given placebo. Otherwise, the treatment for the placebo group patients was the same as for the OAC group. Every 3 months, we measured serum calcium, phosphorus, parathyroid hormone, albumin, and FGF-23 levels. The end point of this phase was the development of hyperphosphatemia. Patients with hyperphosphatemia entered the second phase. All patients entering the second phase underwent coronary artery multidetector computed tomography (MDCT), and serum specimens were taken. Then patients were randomly divided into three groups: the oral activated charcoal (OAC) group, the calcium carbonate (CC) group and the lanthanum carbonate (LC) group. The OAC group was given oral activated charcoal three times a day. When serum calcium was less than 8.4 mg/dl, calcium carbonate was given. When necessary, active vitamin D was given at the same time, to maintain blood calcium levels between 8.4 and 9.6 mg/dl. Other treatment options remained unchanged. The CC and LC groups were given calcium carbonate and lanthanum carbonate, respectively, according to the Kidney Disease: Improving Global Outcomes (KDIGO) and Chronic Kidney Disease–Mineral and Bone Disorder (CKD–MBD) formula. The therapeutic schedule remained unchanged during the study after patients entered the CC or LC group. Coronary MDCT was performed every 6 months to evaluate vascular calcification score.

Specimen collection and preservation: 8 ml of fasting venous blood sample was collected, and complete blood count, renal function, electrolyte, and intact parathyroid hormone (iPTH) (iPTH Kit, Roche, Germany) were measured in our hospital clinical laboratory. Another 4 ml of blood was centrifuged at 3000 r/min for 10 min, and the supernatant fluid was stored in a − 80° freezer for determination of FGF-23 by enzyme-linked immunosorbent assay (FGF-23 Kit, Bio-Swamp, America).

Vascular calcification score determination (Agatston score): All subjects underwent coronary artery MDCT using a Philips Helical CT scanner. Scanning parameter: voltage: 120 kV, current: 55 mAs, collimation: 32 × 0.625 mm, dose-length product (DLP): 62.4 mGy cm, CTDIvol: 4.5 mGy, rotation time: 0.4 s, no. of cycles: 7. Four coronary arteries (left main trunk, anterior descending artery, circumflex branch, and right coronary artery) were scanned, and calcifications were calculated using imaging software. We used 130 Hu as the threshold, and the calcification was divided into four grades according to the CT values: CT values of 130–199 Hu were designated grade 1, CT values of 200–299 Hu were designated grade 2, CT values of 300–399 Hu were designated grade 3, and CT values of 400 Hu and above were defined as grade 4. The sum of the calcification scores for all four coronary artery branches were calculated to obtain the total coronary artery calcification score (CACS).

*Endpoint* Loss to follow-up, dialysis treatment and loss of contact were considered as failure to complete tests. The primary endpoint of the first phase was the development of hyperphosphatemia, which was defined as serum phosphorus levels greater than 5.8 mg/dl on two consecutive fasting blood tests at the end of 12-month follow-up. The end point of the second phase was the development of vascular calcifications in patients without vascular calcifications before enrollment or doubling of calcification scores in patients who had vascular calcifications prior to enrollment at the end of 12-month follow-up.

### Statistical methods

SPSS 19.0 statistical software was used for data analyses. Normally distributed measurement data were expressed as the mean ± standard deviations or, in situations where distributions were skewed, as median and interquartile range. Interquartile range was defined as the difference between the median of quartile 1 and the median of quartile 3. The independent samples *t* test, one-way ANOVA and Kruskal–Wallis test were used to perform statistical comparisons between groups. The LSD method was used to compare differences between groups. Kaplan–Meier survival curves were drawn, and survival time of different groups was compared using the log-rank test. P < 0.05 was considered statistically significant.

For phase 1,the following formula was used in the study: N = 〖2×[(µ_α + µ_β)σ/δ]〗^2α = 0.05 (1 tail), β = 0.1, µ_α = 1.64 (1 tail), µ_β = 1.28 (1 tail), σ = 0.6 [1], δ = 0.5, N = 26, total n = 52. For phase 2, we used PASS11 software to calculate the sample size, and the three sets of means were LC = 616, CC = 755, OAC = 525, respectively. The standard deviation was 180, α = 0.05 (2 tail), β = 0.15. The sample size of each group was 15, total n = 45. In this trial, we used the method of stratified randomization and sequentially numbered opaque, sealed envelopes.

## Results

### Subject characteristics

*General information* According to the inclusion and exclusion criteria, a total of 97 patients were included in the study, including 50 males and 47 females, aged 56.9 ± 16.5 years. The basic characteristics of the research patients are shown in Table 1, the reasons for patients exclusion form study were reported in Fig. 1.

**Table 1:** The basic characteristics of the research patients

General characteristics	oAC group	Placebo group	*t*/*X*^2^	P value
Gender (male/female)	25/24	25/23	0.011	0.917
Age ($\bar {x}$ ± s)	57.0 ± 16.7	56.8 ± 15.8	0.067	0.861
Chronic kidney disease causes				
Chronic glomerulonephritis	19/49	19/48	0.007	1.000
Diabetic nephropathy	16/49	15/48	0.022	1.000
Hypertensive kidney damage	7/49	6/48	0.067	1.000
Drug-induced renal damage	3/49	4/48	0.177	0.715
Polycystic kidney	2/49	1/48	0.323	1.000
Other	2/49	2/48	0.000	1.000
Calcium (mg/dl)	9.49 ± 0.44	9.49 ± 0.38	− 0.052	0.959
Phosphorus (mg/dl)	3.74 ± 0.46	3.90 ± 0.41	− 1.785	0.077
iPTH (mmol/l)	132.27 ± 45.65	133.28 ± 45.65	− 0.111	0.912
FGF-23 (pg/ml)	243.73 ± 26.45	243.44 ± 26.72	0.055	0.956
CACS	34.35 ± 26.17	35.79 ± 23.94	− 0.284	0.777

**Fig. 2:**
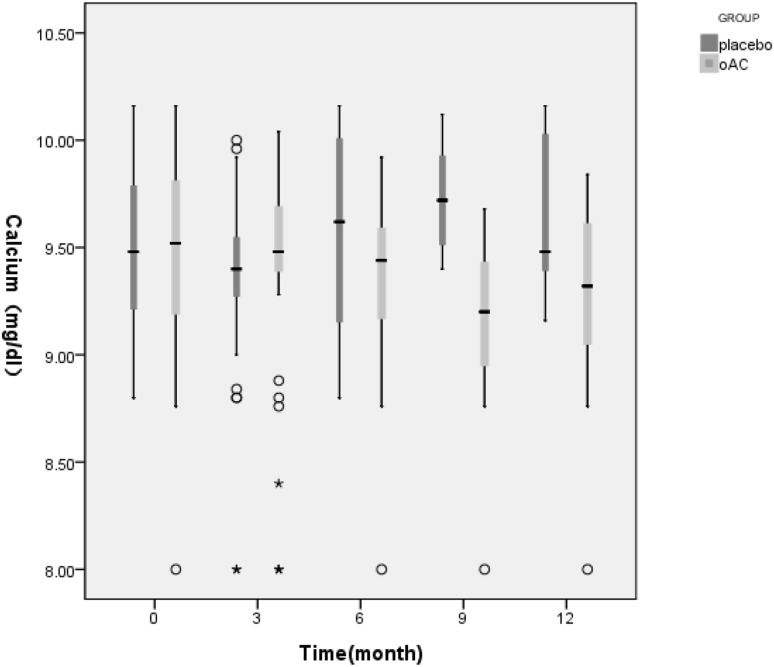
Box figure showing serum calcium level changes in the first phase of the experiment. White circle: outliers

**Fig. 3:**
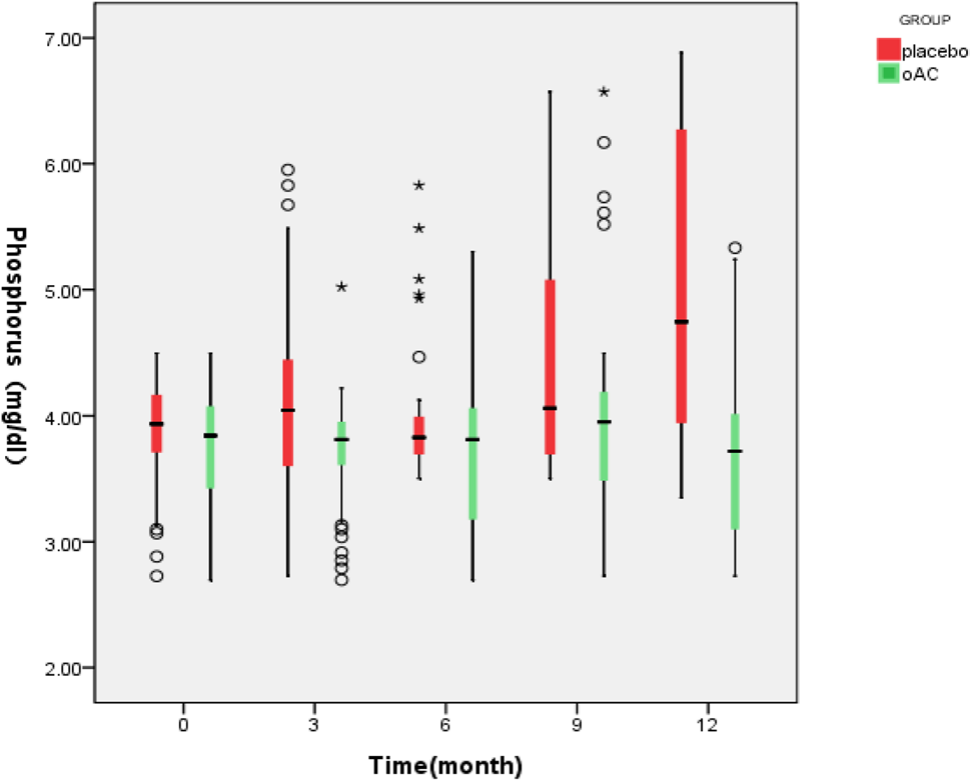
Box diagram showing changes in serum phosphorus levels in the first phase of the study. White circle: outlier

**Fig. 4:**
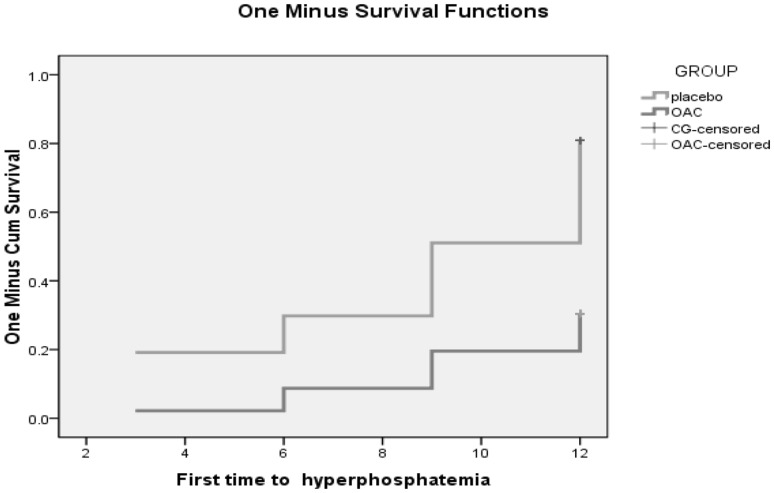
Kaplan–Meier survival curves of the first phase

The first phase of the follow-up is 12 months. Figures 2 and 3 showed that in the first phase of the experiment, there was no significant change in serum calcium values between the OAC group and the placebo group (t = − 1.765, P = 0.083). However, beginning from the 3rd month, the phosphorus levels of the OAC group started to become lower than that of the placebo group, and remained so until the end of the experiment (t = 3.653, P = 0.000). This difference was maintained until the end of the first phase of the trial. Also, there was a statistically significant difference in the percentage of hyperphophatemia patients between the OAC and placebo groups (28.57% vs. 79.17%, X^2^ = 24.958, P = 0.000). According to Fig. 4, we noticed that the OAC group had a survival advantage over the placebo group related to delayed onset of hyperphosphatemia (P < 0.05) (Table 2).

**Table 2:** The change of CACS value in the second stage of the study

Follow-up time (month)	CACS			*F*	Sig.
	oAC	CC	LC		
0	210.4 ± 80.9	189.9 ± 103.7	186.9 ± 80.0	0.346	0.709
6	246.4 ± 68.3^a^	362.5 ± 105.6	197.7 ± 72.0	16.952	0.000
12	426.0 ± 100.8^b^	526.1 ± 71.0	338.8 ± 134.9	13.195	0.000
18	483.3 ± 102.0^c^	579.9 ± 85.6	357.9 ± 146.7	15.407	0.000
24	525.5 ± 104.2^d^	688.1 ± 183.7	431.4 ± 122.5	13.006	0.000

OAC group compared with LC group, ^a^*P* > 0.05, ^b^*P* < 0.05, ^c^*P* < 0.01, ^d^*P* > 0.05

**Fig. 5:**
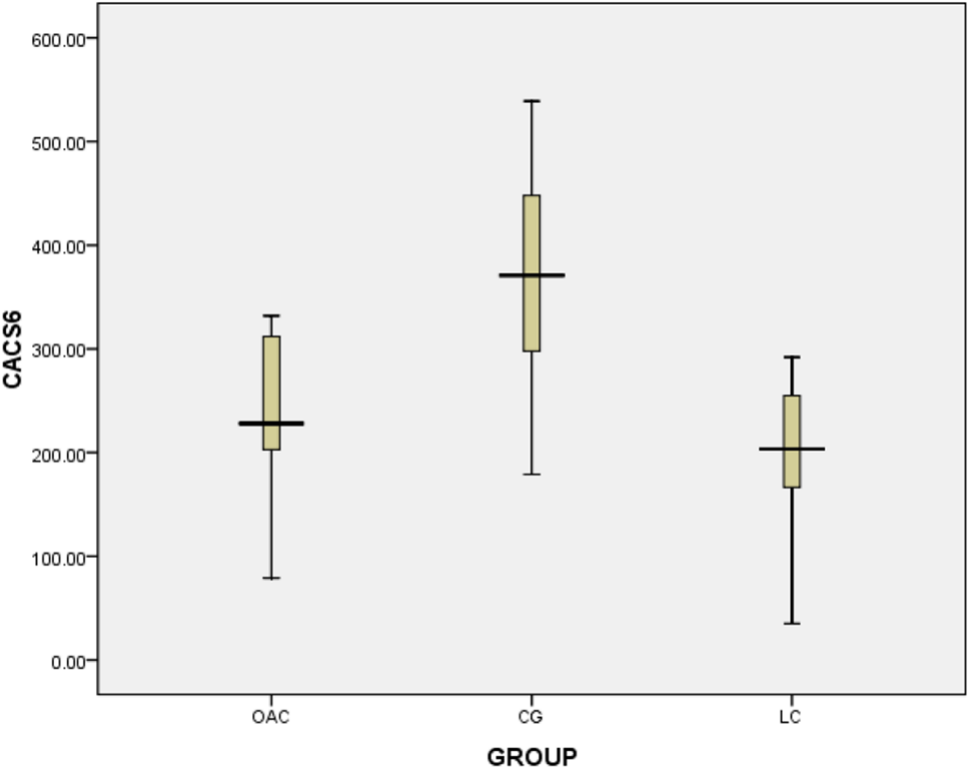
Box diagram showing CACS values at 6 months

**Fig. 6:**
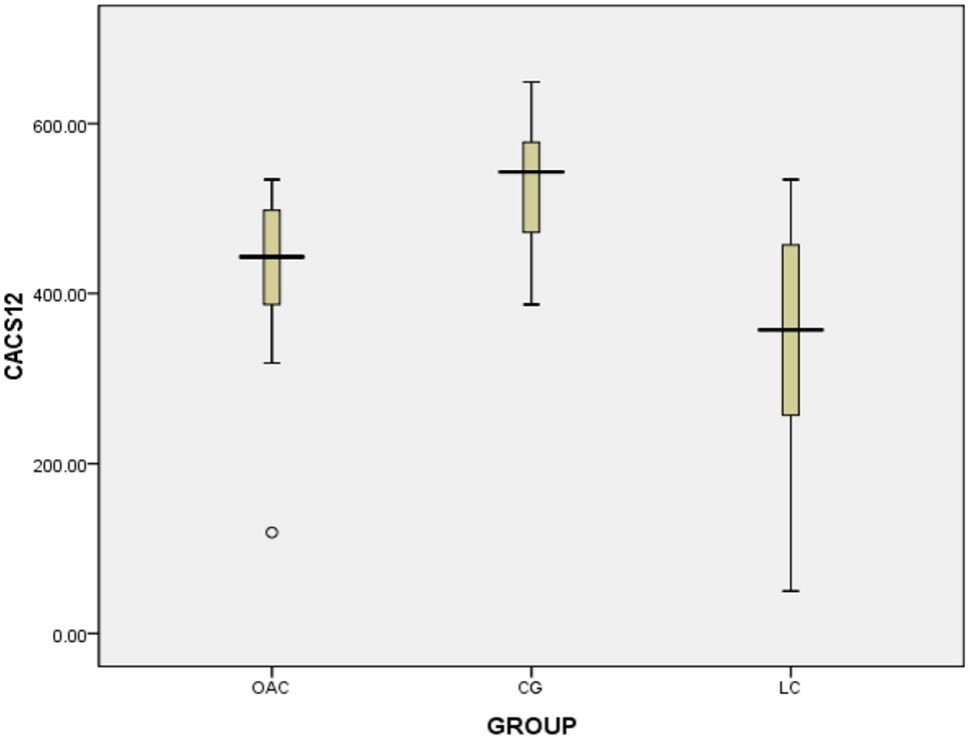
Box plot showing CACS values at 12 months. White circle: outlier

**Fig. 7:**
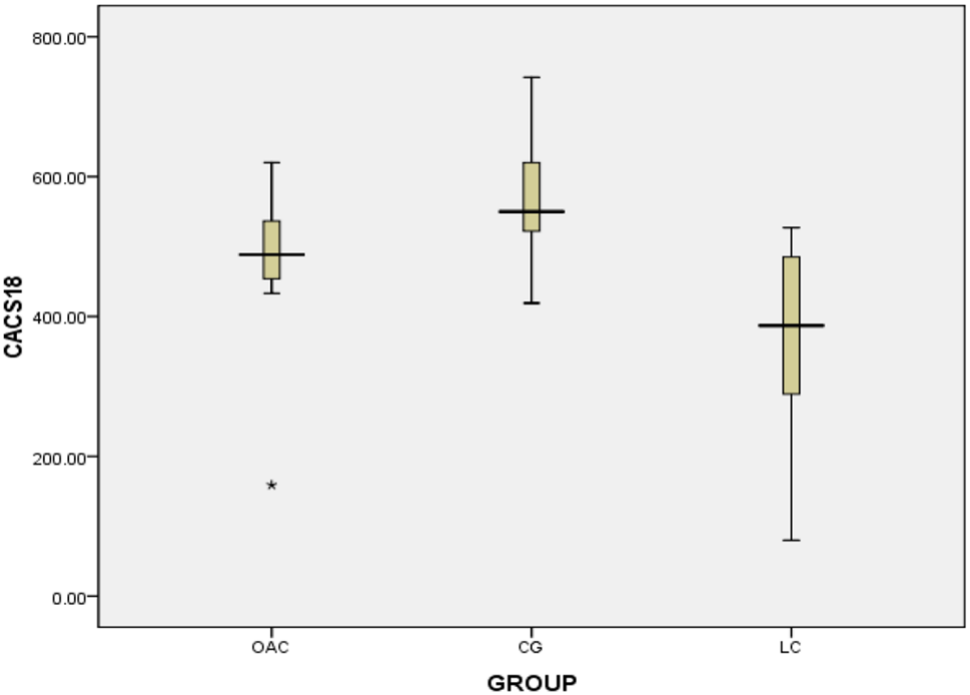
Box plot showing CACS values at 18 months. Black star: extreme value

**Fig. 8:**
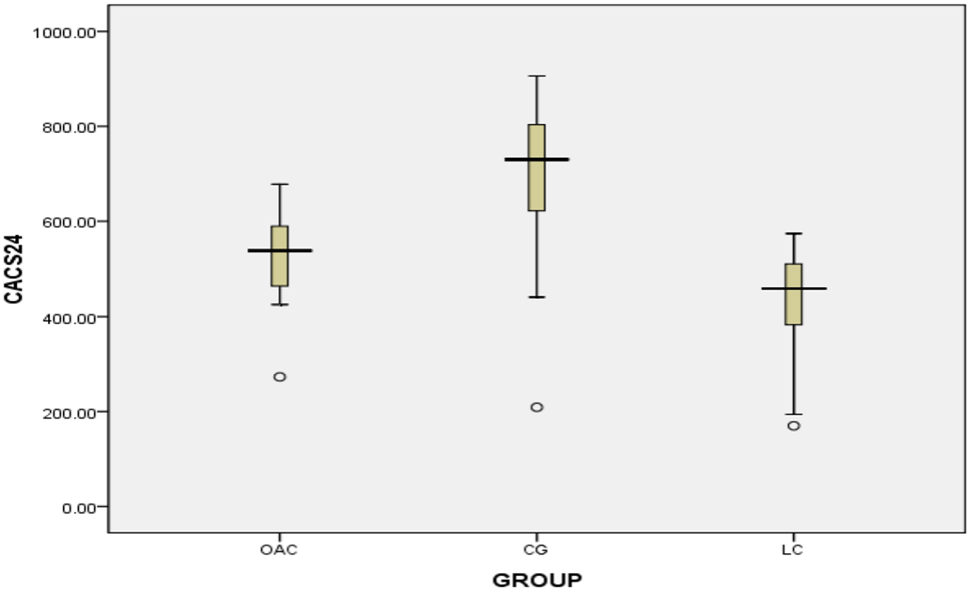
Box plot showing the CACS at 24 months. White circle: outlier

**Fig. 9:**
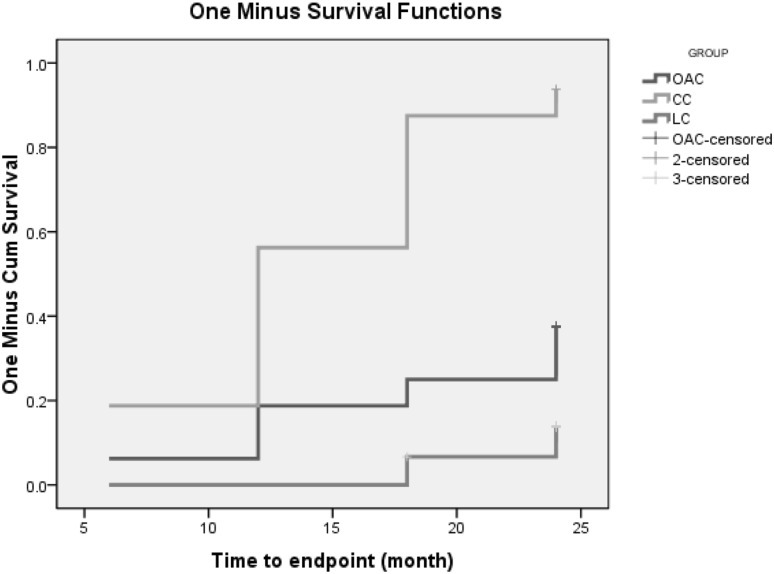
Kaplan–Meier survival curve of the second phase of the study

The second phase of the follow-up lasted 24 months. At the end of the trial we found that five patients were assigned to OAC group in the first phase and then to OAC group in the second phase. There was no washout period between phase 1 and phase 2. Figures 5, 6, 7 and 8 showed that in the 6th months, the differences in CACS between the OAC group, the CC group and the LC group were statistically significant (246.4 ± 68.3 vs 362.5 ± 105.6 vs 197.7 ± 72.0, P < 0.01). However, there was no significant difference between the OAC group and the LC group (P > 0.05). By the 12th month, the differences in CACS between the OAC group, the CC group and the LC group were statistically significant [443.0 (386.0–501.0) vs 543.0 (471.5–580.0) vs 357.0 (246.5–457.0), P < 0.01]. There were significant differences between the OAC and LC groups (P < 0.05). By the 18th month, the differences in CACS between the OAC group, the CC group and the LC group were statistically significant [488.5 (445.0–538.3) vs. 550.0 (522.0–650.0) vs 387.0 (238.0–491.0), P < 0.01]. There were significant differences between the OAC and LC groups (P < 0.01). By the 24th month, the differences in CACS between the OAC group, the CC group and the LC group were significant (525.5 ± 104.2 vs 688.1 ± 183.7 vs 431.4 ± 122.5, P < 0.05), while there was no significant difference between the OAC group and the LC group (P > 0.05). According to Fig. 9, we noticed that the OAC and LC groups were superior to the CC group in preventing the onset of vascular calcifications (P < 0.01). There was no significant difference between the OAC and LC groups (P > 0.05).

There were 20 episodes of adverse events in 7 (10.6%) patients during the OAC treatment. Most of the adverse events were minor to moderate gastrointestinal disorders, such as constipation and abdominal distention or pain. Six of these patients were treated with oral laxative or anti-dopaminergic drug and were able to complete the study. One patient was withdrawn from the study due to severe constipation and recovered subsequently by taking oral laxative. Also, there were three patients developed abdominal distension and hernia during the CC group and two patients developed mild diarrhea during the LC group.

## Discussion

In recent years, delaying the progression of CKD, extending the life expectancy of patients with chronic kidney disease, and improving their quality of life have become the focus of clinical work of clinical nephrologists. CVD is one of the major complications of CKD patients, accounting for 50% of the total mortality [4]. The risk of death in adult patients with CKD is 20–30 times greater than that of the general population without CKD. In addition to traditional risk factors such as age, hypertension, impaired glucose tolerance, and dyslipidemia affecting prognosis, non-traditional risk factors including vascular calcification and oxidative stress have become the focus of research, gaining increasing interests among investigators [5]. Vascular and valve calcification are important risk factors for increasing risk of cardiovascular events and mortality in patients. Many patients have extensive vascular calcifications prior to a cardiovascular event. Vascular and valve calcification in patients is accelerated and aggravated compared to the general population [6].

Our first phase of the pilot study found that OAC can effectively delay the onset of hyperphosphatemia in patients with CKD. Compared with the placebo group, OAC significantly reduced the patient’s serum phosphorus levels. Hyperphosphatemia is a common presence in CKD patients, and is treated by dietary measures, dialysis techniques and/or phosphate binders [7]. Previous studies have shown that phosphate levels above 4.2 mg/dl are associated with increased mortality [8]. Our Kaplan–Meier survival curves also showed that the OAC group had a survival advantage over the control group in preventing hyperphosphatemia. This is consistent with previous studies which showed that increased phosphorus increases the risk of death: for every increase in phosphorus by 1.28 mg/dl, there is a relative 6% increase in the risk of death [9]. Phosphate binders currently available for clinical use in China are limited, and almost all calcium-containing preparations, including calcium carbonate, often lead to hypercalcemia and increase the incidence of cardiovascular disease. This limits the effectiveness of controlling serum phosphorus in patients with chronic renal failure. Oral activated charcoal is a new and effective therapeutic agent, which is an adsorbent of carbonized polymer composite material. It is carbonized at 1100 °C without oxygen or digestion. The prototype is excreted in the feces and has the characteristics of a 1000–1400 m^2^ specific surface area per gram of medical charcoal. Activated charcoal has a strong adsorption force, with various pore sizes at the surface. After ingestion, it rapidly spreads in the intestine, strongly absorbing small molecules and toxic substances [10]. It has a role in reducing blood phosphorus level. Some researchers noted that oral activated charcoal can effectively reduce the level of serum phosphorus, serum urea and creatinine and can be clinically applied safely, with few side effects [11]. Lanthanum carbonate is a calcium-free phosphate-binder, which in addition to effective phosphate binding offers the advantage of a low pill burden [12]. Lanthanum ions have a high affinity for phosphate, and combined with lanthanum phosphate, lanthanum carbonate is less soluble in water and is excreted from the body. With oral ingestion of lanthanum, 80% is excreted in bile and about 13% from the intestinal tract, indicating that lanthanum excretion does not depend on renal function [13]. At present, studies have confirmed that the efficacy and safety of lanthanum carbonate treatment are superior to calcium carbonate [14]; results of the second phase of our study supported this point of view.

Patients who developed hyperphosphatemia entered the second phase of our trial. Previous studies have shown that assessment of abdominal aortic calcification (AAC) in CKD patients can help identify patients at higher cardiovascular risk and may provide important information for personalized treatment. We aimed to investigate changes in CACS in patients with stage 3–4 CKD [15]. Follow-up of phase II patients showed that there was a significant difference in CACS between the OAC group and the CC group, but no significant difference between the CC and LC groups, or the OAC and LC groups. Kaplan–Meier survival curves showed that the OAC and LC groups were superior to the CC group in preventing vascular calcification, while there was no significant difference between the OAC and LC groups. The average value of CACS among the three groups was highest in the CC group, followed by the OAC group and the LC group, indicating that medical charcoal has the effect of delaying the development of vascular calcification in non-dialysis patients with CKD. Previous studies showed that hyperphosphatemia was a major risk factor for vascular calcification in patients with CKD [16]. Its mechanism may include many aspects. First, increased blood phosphorus can promote vascular smooth muscle cell (VSMC) conversion to an osteoblast-like phenotype, primarily by the contraction and contraction function of the systolic phenotype. As a synthetic phenotype, the type III sodium-phosphate transporter (Pit-1) was activated in the type III-phosphate co-transporter (NPC) on the cell membrane of VSMCs in high phosphorus conditions. At higher concentrations than normal, Pit-1 can transmit signals to the cells, eventually leading to VSMC transdifferentiation and calcification of osteocytes [17]. Second, elevated serum phosphorus enters cells through the sodium-phosphorus co-transporter Pit-1 [18], leading to an increase in intracellular phosphorus levels. When intracellular levels of phosphorus are elevated, expression of nuclear transcription factors such as the runt-related transcription factor (Runx2) can be induced to initiate the vascular calcification process. This phenotypic switch plays a very important role in vascular calcification [19]. Third, VSMCs are easily damaged in high-phosphorus environments. As the release of matrix vesicles increases, a large number of vesicles are dissolved, leading to the rapid rise of calcium ion concentration, thereby accelerating cell apoptosis, forming calcification nests, and initiating calcification [20]. The fact that VMSC apoptosis may occur earlier than the occurrence of calcification is an important part of vascular calcification caused by hyperphosphatemia [21]. Fourth, hyperphosphatemia can promote VSMC extracellular matrix remodeling. For VSMCs in a high-phosphorus environment, the surface has apatite-containing matrix vesicles and calcified collagen fibers. These matrix vesicles provide sites for further calcification. High-phosphorus stimulation increases the secretion of matrix vesicles, thereby aggravating vascular calcification [22, 23].

## Conclusion

OAC effectively delays the onset of hyperphosphatemia in patients with chronic kidney disease. OAC appears to delay the development of vascular calcifications in stage 3–4 CKD patients.

At present, there are few studies regarding the prevention of vascular calcification in patients with CKD. We observed and explored the effects of OAC on hyperphosphatemia and vascular calcification in patients with stage 3–4 CKD. There are several limitations to this study: it is a single-center clinical study, with a limited number of cases, and the observation time was short, especially in the CKD sub-group analysis. The small number of cases may affect the statistical analysis. Therefore, our conclusions should be studied in-depth in the future.

## Electronic supplementary material

Below is the link to the electronic supplementary material.

Supplementary material 1 (DOCX 44 KB)
