# Supplementary material for: Effects of oral activated charcoal on hyperphosphatemia and vascular calcification in Chinese patients with stage 3–4 chronic kidney disease
Source: J Nephrol. 2018 Dec 26;32(2):265–72. doi: 10.1007/s40620-018-00571-1 (PMC6422957; doi:10.1007/s40620-018-00571-1)
Supplement: Supplementary file 1 — Supplementary material 1 (DOCX 44 KB) [file 40620_2018_571_MOESM1_ESM.docx]

1.For phase 1,the formula was used in the study: N=〖2×[(μ_α+μ_β )σ/δ]〗^2α = 0.05 (1 tail), β = 0.1μ_α=1.64 (1 tail), μ_β=1.28 (1 tail), σ=0.6[1], δ=0.5,N = 26, total n=52.

For phase 2, We used PASS11 software to calculate sample size, and the three sets of means were respectively LC=616,CC=755,OAC=525, the standard deviation is 180, α = 0.05(2 tail), β=0.15.The sample size of each group was 15, total n=45.

2. We use the method of stratified randomization and sequentially numbered, opaque, sealed envelopes.

The specific method is shown in the figure and table.

The first phase as the table:

|  | **oAC group** | **Placebo group** |
| --- | --- | --- |
| Male, age18～40, CKD3 | 2 | 2 |
| Male, age40～60, CKD3 | 5 | 5 |
| Male, age＞60, CKD3 | 5 | 5 |
| Male, age18～40, CKD4 | 3 | 3 |
| Male, age40～60, CKD4 | 6 | 6 |
| Male, age＞60, CKD4 | 4 | 4 |
| Female, age18～40, CKD3 | 2 | 2 |
| Female, age40～60, CKD3 | 5 | 5 |
| Female, age＞60, CKD3 | 4 | 4 |
| Female, age18～40, CKD4 | 3 | 3 |
| Female, age40～60, CKD4 | 6 | 5 |
| Female, age＞60, CKD4 | 4 | 4 |


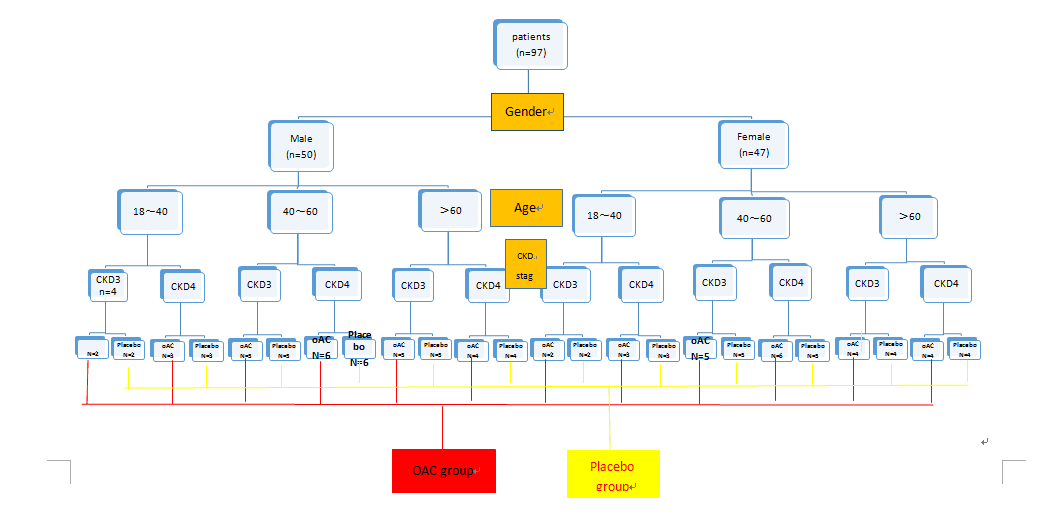


The second phase as the table:

|  | **oAC** | **CC** | **LC** |
| --- | --- | --- | --- |
| Male, age18～40, CKD3 | 1 | 1 | 1 |
| Male, age40～60, CKD3 | 2 | 2 | 2 |
| Male, age＞60, CKD3 | 1 | 1 | 1 |
| Male, age18～40, CKD4 | 2 | 2 | 2 |
| Male, age40～60, CKD4 | 3 | 2 | 2 |
| Male, age＞60, CKD4 | 1 | 1 | 1 |
| Female, age18～40, CKD3 | 0 | 0 | 0 |
| Female, age40～60, CKD3 | 2 | 2 | 2 |
| Female, age＞60, CKD3 | 1 | 1 | 1 |
| Female, age18～40, CKD4 | 1 | 1 | 1 |
| Female, age40～60, CKD4 | 2 | 3 | 2 |
| Female, age＞60, CKD4 | 1 | 1 | 1 |


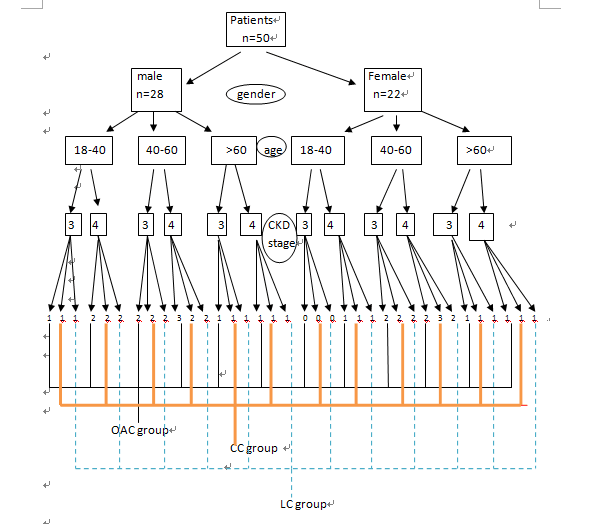


3.

**Table 3: The change of CACS value in the second stage of the study**

| **Follow-up time（month）** |  | **CACS** | | |  | ***P* value** |
| --- | --- | --- | --- | --- | --- | --- |
|  |  | **oAC** | **CC** | **LC** |  |  |
| **0** |  | **210.4±80.9**  **205.0（167.0-287.0）** | **189.9±103.7**  **176.0（107.5-280.5）** | **186.9±80.0**  **185.0（131.3-247.0）** |  | **0.709** |
| **6** |  | **246.4±68.3^a^**  **228.0（202.5-317.0）** | **362.5±105.6**  **371.0（276.0-450.5）** | **197.7±72.0**  **203.5（164.3-261.0）** |  | **0.000** |
| **12** |  | **443.0（386.0-501.0）^b^** | **543.0（471.5-580.0）** | **357.0（246.5-457.0）** |  | **0.000** |
| **18** |  | **488.5（445.0-538.3）^c^** | **550.0（522.0-650.0）** | **387.0（238.0-491.0）** |  | **0.000** |
| **24** |  | **525.5±104.2^d^**  **538.0（437.0-549.0）** | **688.1±183.7**  **730.0（616.0-806.8）** | **431.4±122.5**  **458.0（360.0-530.0）** |  | **0.000** |

Results are reported as mean ± standard deviation for normally distributed variables or median (interquartile range) for variables with skewed distributions.

Note: OAC group compared with LC group, *^a^P>* 0.05, *^b^P <*0.05, *^c^P <*0.01, *^d^P >* 0.05.
